# Genetic lesions within the 3a gene of SARS-CoV

**DOI:** 10.1186/1743-422X-2-51

**Published:** 2005-06-20

**Authors:** Timothy HP Tan, Timothy Barkham, Burtram C Fielding, Chih-Fong Chou, Shuo Shen, Seng Gee Lim, Wanjin Hong, Yee-Joo Tan

**Affiliations:** 1 Institute of Molecular and Cell Biology, 61 Biopolis Drive, 138673 Singapore; 2 Tan Tock Seng Hospital, 11 Jalan Tan Tock Seng, 308433 Singapore

## Abstract

A series of frameshift mutations within the 3a gene has been observed in culture-derived severe acute respiratory syndrome coronavirus (SARS-CoV). We report here that viral RNA from clinical samples obtained from SARS-CoV infected patients also contains a heterogeneous population of wild-type and mutant 3a transcripts.

## Findings

### Introduction

Numerous isolates of the severe acute respiratory syndrome coronavirus (SARS-CoV) have been completely sequenced [1-4]. In most cases, only synonymous or non-synonymous substitutions have been reported within the major viral genes, which include replicase 1a/1b gene products, spike, membrane, nucleocapsid and envelope [1-4]. On the other hand, large insertions or deletions have been found in the part of viral genome that encodes the SARS-CoV accessory proteins which have no viral homologues. The ORF 8a/8b region appears to be particularly prone to mutations as deletions of up to 415 bp have been observed in some isolates [2]. Although these mutations do not appear to have any adverse effect on the survival of the virus, it is conceivable that these mutations may have effects on viral pathogenesis *in vivo*, as have been observed for other coronaviruses [5].

We have previously reported that a frameshift mutation occurs within the 3a gene of culture-derived SARS-CoV, which results in a protein with a distinctively shorter N-terminus than the wild-type form [6]. Protein 3a is one of the SARS-CoV accessory proteins and the expression of the 3a protein has been demonstrated during both *in vitro *and *in vivo *infection [5]. To determine if the mutation arises from repeated passages of the virus or if the mutation exists in the virus that is replicating in SARS-CoV infected patients, we analyzed viral RNA isolated directly from 8 clinical samples and determined the sequence of the 3a gene. Interestingly, we have found evidence of a heterogeneous population of subgenomic RNA 3 (sgRNA3) transcripts in patients with acute SARS-CoV infection containing copies of wild-type and mutant 3a genes.

### The Study

Total RNA was extracted from 8 patients confirmed with SARS-CoV infection, as defined by WHO guidelines. The use of clinical samples for this study was approved by the Tan Tock Seng Hospital ethics committee. Reverse transcription (RT, Superscript II RT, Invitrogen) was performed on all samples, according to the manufacturer's protocol, and was followed up by a nested polymerase chain reaction (PCR). The PCR conditions and subsequent cloning steps have been described elsewhere [6]. Essentially, 15 independent clones from each of the eight SARS patient samples were sequenced. As a polymerase fidelity control of the RT and PCR system, full-length 3a RNA was *in vitro *transcribed from pXJ40-3a, a cDNA construct for expressing 3a in mammalian cells [6], and subjected to an identical follow-up PCR and cloning protocol.

For the fluorescence-activated cell sorting (FACS) and Western blot analysis, Vero E6 cells were transiently transfected with pXJmyc-GST, pXJmyc-3a or pXJmyc-3amut1 as previously described (3a and 3amut1 are also known as U274 and U274mut1, respectively, in ref. 6). All these constructs were tagged with the c-*myc *epitope at the N-terminus. All these experiments were performed as previously described [6].

### Results and Conclusions

We have previously identified an oligo(T) tract within the 3a gene, located 16 bp after the first ATG initiation codon, which is prone to insertional mutations [6]. According to several analyses of about 100 genomic sequences of SARS-CoV isolates (in total) obtained from human and animal populations and that have been deposited with Genbank, there has been no report of mutations in this region [1-4]]. It is possible that direct sequencing results do not show this mutation if it is present in only a minority population. There was a previous report on a frameshift mutation in the 3a gene but the identity of this isolate was not mentioned [7]. The 3a gene from culture-derived SARS-CoV isolates contained heterogeneous extensions at this internal oligo(T) tract. The nature of this extension was such that up to three additional T's were added to the 6T's tract. Any change in the number of T's in this oligo(T) tract, other than in multiples of three, would result in a frameshift mutation and premature translation termination of 3a.

**Figure 1:**
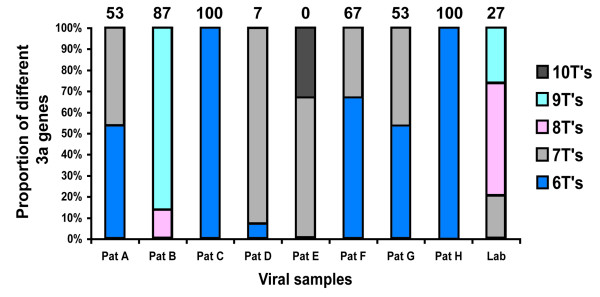
**Distribution of 3a sequence variants within a population of SARS-CoV obtained from clinical and culture-derived samples**. Pat A to H: patients confirmed with acute SARS-CoV infection. Lab: Vero E6 cells infected with SARS-CoV. The value above each column represents the percentage of in-frame 3a genes within the population.

We analyzed this region of the 3a gene from eight patients confirmed with SARS-CoV infection and have detected the presence of sgRNA3 transcripts, carrying 6T's to 10T's tract, in these patients (Figure 1). As our polymerase fidelity controls have confirmed that it is highly unlikely that sequencing and PCR errors are the source of these nucleotide aberrations (data not shown), and these results showed that different variants of the 3a gene exist in the viruses that were replicating in these patients. The percentage of the different mutant transcripts varied considerably from patient to patient. However, in 6 out of the 8 patients, more than 50 % of the sgRNA3 transcripts contains either 6T's or 9T's, which means that the full-length 3a (or with 1 additional amino acid) will be expressed. In patient D, less than 10 % of the transcripts are in-frame, while in patient E, none of the transcripts is in-frame, indicating that the full-length 3a protein will be expressed at a low level (Pat D) or not expressed at all (Pat E). In comparison, about 27 % of the transcripts from a culture-derived virus are in-frame [6]. Overall, these results showed that the frameshift mutations of the 3a gene are not specific to culture-derived SARS-CoV and that they point towards the existence of quasispecies within a given population of SARS-CoVs. As deduced from their study of sequence variation of the spike gene from viral isolates, another group has also suggested that SARS-CoV quasispecies exists *in vivo *[8].

**Figure 2:**
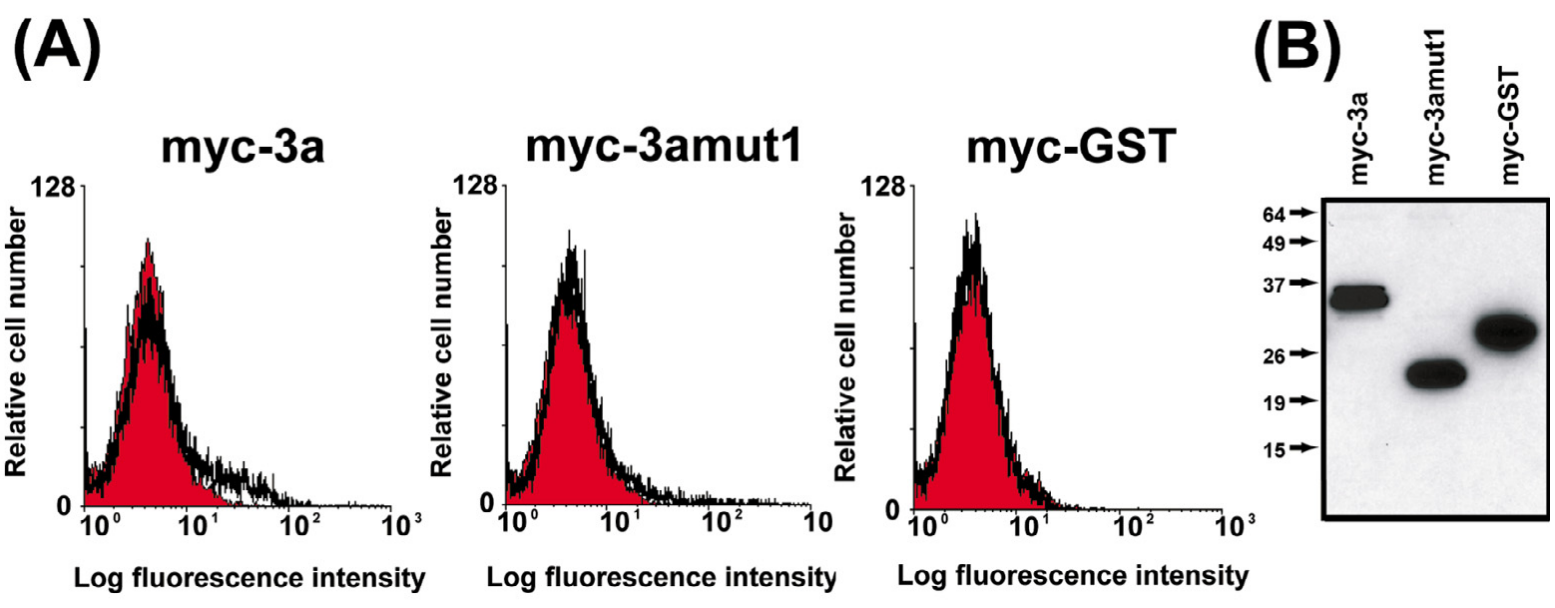
**Detection of myc-3amut1 in transfected Vero E6 cells by FACS and Western blot analysis**. (A) FACS analysis of live cells transfected with myc-3a, myc-3amut1 and myc-GST. Cells were initially probed with anti-myc monoclonal antibodies followed by the corresponding FITC-conjugated antibody. (B) Expression levels of the myc-tagged proteins in cells used for the FACS experiment were determined by Western blot analysis. Probing was done with anti-myc polyclonal antibody.

Translation of a frameshifted 3a gene would terminate shortly after 18 residues or so. Nonetheless, there are two alternative initiation codons located downstream. Translation from these would result in two possible smaller 3a gene products. We have shown that at least one of these truncated forms of 3a, named as 3amut1, can be detected in the lysate of infected cells [6]. It has been shown that the 3a is localized to the cell surface of SARS-CoV infected cells [6,9]. FACS analysis showed that while the myc-3a protein (full-length 3a with a c-*myc *epitope at the N-terminus) was transported to the cell surface in transiently transfected Vero E6 cells, the myc-3amut1 (truncated 3a with the same c-*myc *epitope) could not be detected on cell surface (Figure 2A). Western blot analysis was also performed to ensure that the expressions of the full-length and truncated 3a proteins in the transfected cells were comparable (Figure 2B). As 3amut1 corresponds to 101 to 274 amino acids of 3a and lacks the first two transmembrane domains [6], our results showed that these two transmembrane domains are essential for the expression of 3a on the cell surface.

It is possible that the aberrant nature of the oligo(T) tract in the 3a gene is akin to that of the nucleotide insertion event in the oligo(A) tract of the 3b gene of infectious bronchitis virus (IBV), another coronavirus [10]. The insertion of a single adenylate nucleotide into this region of 3b resulted in a C-terminally truncated gene product. The corresponding mutant 3b was also localized differently within the cell. In view of the fact that 3a has also been shown to interact with the spike protein [6,7] and that both proteins have a tendency to co-mutate [7], it would be interesting to know whether these serial frameshift mutations can also be correlated with a recognizable mutation pattern of the spike gene.

In other RNA viruses, such as the foot-and-mouth disease virus (FMDV) and human respiratory syncytial virus, internal poly(A) extensions have been identified before as a hot spot for mutations [11,12]. Similarly, these extensions also create frameshift mutations in the affected genes. FMDV populations with longer poly(A) extensions seem to have a lower fitness value as compared to those with shorter extensions [11]. This is despite the fact that both the wild-type and mutant forms of the affected FMDV protein, the L protease, have equal functionality [13]. In addition, viral genomes possessing different lengths of the poly(A) tract could be recognized even from just a single PFU of FMDV [11].

It is intriguing to find that unlike 3a, the 3amut1 is not transported to the cell surface as the cell surface expression (and endocytotic properties) of 3a may be involved in modulating the trafficking properties of the spike protein [14]. Our results showed that the viruses in some of the patients appear to encode only for the truncated form(s) of 3a and not the full-length 3a protein (Figure 1) and indicated that the functionality of full-length 3a is not essential for virus replication. However, it is also conceivable that the different variants of 3a have different stabilities and/or functions, and hence would contribute differently to viral pathogenesis *in vivo*. In addition, it was recently reported that 3a is a structural protein and at least 2 truncated forms of 3a were dominantly present in the virion [9]. Further studies will reveal if the truncated forms of 3a, which results from frameshift mutations in the viral genome, can be incorporated in the virion and if there are phenotypic effects of a truncated 3a during the infection cycle. With respect to viral viability in the natural host, does full-length 3a confer a fitness gain over truncated 3a? If so, the possibility remains that under selective pressure, the distribution of viral genotypes could tip in favor to those which carry the wild-type 3a gene. A similar genotypic reversion event has been documented for FMDV [11]. Further studies on a larger cohort of patients will be necessary to establish if there is a relationship between the mutations observed in the 3a transcripts and the severity of the clinical symptoms in individual patients.

## Competing interests

The author(s) declare that they have no competing interests.

## Authors' contributions

THPT carried out all experimental work and drafted the manuscript. YJT conceived of the study and helped to draft the manuscript. TB provided the clinical samples. BCF, CFC, SS, SGL and WH also assisted THPT and YJT in drafting the manuscript. All authors read and approved the final manuscript.
